# Comparison of Audiometric Outcomes Following Acute Labyrinthitis

**DOI:** 10.3390/medicina61122083

**Published:** 2025-11-22

**Authors:** Lara Dreu, Maja Gabor, Petra Povalej Bržan, Janez Rebol

**Affiliations:** 1 Faculty of Medicine, University of Maribor, Taborska Ulica 8, 2000 Maribor, Slovenia; lara.dreu333@gmail.com (L.D.); maja.gabor00@gmail.com (M.G.); petra.povalej@um.si (P.P.B.); 2 Faculty of Electrical Engineering and Computer Science, University of Maribor, Koroska Cesta 46, 2000 Maribor, Slovenia; 3 Department of Otorhinolaryngology, Head and Neck Surgery, University Medical Centre Maribor, Ljubljanska Ulica 5, 2000 Maribor, Slovenia

**Keywords:** labyrinthitis, hearing loss, vertigo, audiometry, COVID-19, inner ear

## Abstract

*Background and Objectives*: Labyrinthitis is an inflammatory inner ear disorder often resulting in acute vertigo and hearing loss. While typically self-limiting, some cases lead to persistent deficits. This study examined incidence trends of acute labyrinthitis before and after 2020 and compared hearing outcomes between these periods. *Materials and Methods*: This retrospective cohort study included 126 patients diagnosed with acute labyrinthitis at a tertiary medical center between January 2014 and May 2024. Patients were divided into pre-2020 (2014–2019) and post-2020 (2020–2024) cohorts. Poisson regression analyzed incidence trends, while audiometric outcomes were compared in 79 patients with complete 3-month follow-up data. Hearing recovery was assessed using pure-tone averages (PTA) across 500–4000 Hz, and predictors of persistent impairment were identified through multivariable logistic regression. *Results*: The cohort had a mean age of 47.8 years with female predominance (63.5%). Annual case counts increased significantly post-2020 (19.8 vs. 6.5 cases/year; CRR 3.05, 95% CI 2.07–4.57, *p* < 0.001). Substantial hearing improvement occurred across all frequencies (median AC improvement 13.8 dB). Hearing recovery was comparable between periods, with similar PTA improvements (−16.7 vs. −15.3 dB, *p* = 0.73) and equivalent distributions across World Health Organization (WHO) hearing categories (*p* = 0.64). Baseline hearing level was the strongest predictor of persistent impairment (OR 1.56 per 5 dB increase, 95% CI 1.25–1.95, *p* < 0.001), while age, sex, and diagnostic period showed no significant association. Among post-2020 patients, only 12.6% had confirmed coronavirus disease 19 (COVID-19), and no reliable association with hearing outcomes could be established due to substantial missing data. *Conclusions*: A significant increase in hospital-diagnosed labyrinthitis cases occurred following 2020, yet hearing outcomes at 3-month follow-up remained consistent with the pre-pandemic period. Baseline hearing level was the primary determinant of recovery, unaffected by demographic factors or diagnostic period. These findings suggest that while pandemic-related factors may have influenced case frequency, they did not alter auditory prognosis or recovery patterns.

## 1. Introduction

Labyrinthitis is an inflammatory condition of the inner ear that typically presents with the acute onset of vertigo, nausea, sensorineural hearing loss, and tinnitus. While often self-limiting, a significant proportion of patients experience persistent auditory or vestibular deficits, yet the factors predicting long-term hearing recovery remain incompletely characterized [1,2]. Although comprehensive epidemiological data remain limited, the incidence appears to increase with advancing age [1]. The condition is most commonly caused by viral or bacterial infections but may also occur as a manifestation of systemic autoimmune disease or human immunodeficiency virus (HIV) infection. Viral etiologies are the most frequent, typically developing secondary to an upper respiratory tract infection. These cases are usually self-limiting; however, recurrent episodes may result in permanent sensorineural hearing loss. Reported viral pathogens include influenza, parainfluenza, adenovirus, herpes simplex virus type 1, coxsackievirus, rubella, respiratory syncytial virus (RSV), measles, varicella-zoster, mumps, and cytomegalovirus (CMV). Congenital hearing loss often results from in utero infection with rubella or CMV, whereas mumps and measles remain major postnatal causes. Herpes zoster oticus (Ramsay Hunt syndrome), caused by reactivation of latent varicella-zoster virus, typically presents with a vesicular rash on the auricle or oral mucosa and may be accompanied by facial nerve palsy. The vestibular and cochlear nerves are involved in up to 25% of reactivation cases [1,2,3]. Labyrinthitis typically presents with the acute onset of severe vertigo, nausea, and vomiting. Vertigo may persist continuously for up to 72 h or recur intermittently over several weeks. In contrast to vestibular neuritis, labyrinthitis is frequently accompanied by hearing loss or tinnitus. Nystagmus, with the fast phase directed away from the affected ear, is commonly observed. Clinical balance testing (e.g., Romberg or tandem gait) often reveals postural instability. The Weber and Rinne tests usually indicate sensorineural hearing loss, warranting a comprehensive audiological evaluation. Otoscopic examination may reveal middle ear inflammation or the presence of cholesteatoma [1]. Treatment should be individualized according to the underlying etiology and focused on symptomatic relief. While most patients achieve complete recovery, a subset may experience persistent auditory or vestibulary dysfunction [1,2]. Because several serious conditions—such as cerebrovascular events—can present with similar symptoms, a comprehensive clinical assessment, including detailed history-taking, physical examination, and appropriate diagnostic testing, is essential before establishing the diagnosis [1,4,5,6].

More recently, severe acute respiratory syndrome coronavirus 2 (SARS-CoV-2) infection has been linked to cases of viral labyrinthitis reported during the coronavirus disease 19 (COVID-19) pandemic [7,8,9,10,11,12].

However, establishing a direct causal relationship in a clinical population is profoundly challenging. Widespread testing was not consistently performed, and for many patients, SARS-CoV-2 infection status remains unknown. Therefore, a critical but more feasible question emerged: Did a significant shift in the epidemiology and clinical presentation of labyrinthitis coincide with the pandemic period, regardless of the specific underlying cause?

This study was designed to answer this question by analyzing a single-center cohort over a ten-year period (2014–2024). Its primary aims were (1) to determine whether a notable change in hospital case frequencies occurred after 2020, and (2) to evaluate whether hearing outcomes at a 3-month follow-up differed between the pre- and post-2020 eras.

## 2. Materials and Methods

### 2.1. Study Design and Ethical Considerations

This retrospective cohort was conducted at the Department of Otorhinolaryngology and Head and Neck Surgery of the University Medical Centre Maribor (UKC MB). All collected data were processed and stored anonymously and confidentially, following ethical guidelines for medical research. The study was carried out in line with the Declaration of Helsinki and was approved by the Medical Ethics Committee of the UKC MB. All patient data were anonymized and handled confidentially.

### 2.2. Patient Selection and Diagnostic Criteria

Statistical data processing was performed during the 2024/25 period, and the retrospective analysis covered the years between 20 January 2014, and 23 May 2024.

We reviewed the records of all patients diagnosed with acute labyrinthitis (ICD-10 code H83.0) between January 2014 and May 2024. The diagnosis was made based on the following clinical criteria: (1) a history of acute vertigo lasting at least 24 h, (2) the presence of contralateral nystagmus and impaired balance and (3) audiometrically confirmed unilateral sensorineural hearing loss of at least 30 dB across three consecutive frequencies. Vestibulo-ocular testing was not performed on all patients, which is why we are unable to present the results of vestibular testing. Otomicroscopy was performed on all patients to rule out middle ear pathologies such as acute otitis media and cholesteatoma.

Magnetic resonance imaging (MRI) was performed to exclude retrocochlear pathology for all patients with persistent hearing asymmetry, which we defined as a pure-tone average (PTA) (500, 1000, 2000, 4000 Hz) interaural difference of ≥30 dB at the 3-month follow-up. Based on this criterion, 16 out of the 79 patients in the final cohort underwent MRI.

Patients lacking adequate medical documentation (e.g., missing audiological reports or other essential clinical data), as well as those with other diagnoses that could affect hearing (such as Ménière’s disease or vestibular neuritis without cochlear involvement), were excluded from the analysis.

From the initial cohort of 126 patients, 79 were included in the final analysis with complete audiometric evaluation both at the initial evaluation and at a follow-up visit within three months. The remaining 47 patients were included as a supplementary analysis concerning treatment approaches and SARS-CoV-2 infection status.

Comorbidities were uncommon in the cohort; diabetes mellitus was present in 9 patients (7.1%). Data on autoimmune diseases and smoking history were not systematically collected. Audiometric follow-up was conducted at a median of 92 days (IQR 85-101 days), consistent with the standard 3-month endpoint widely used in sudden sensorineural hearing loss studies to capture the critical window for initial recovery.

### 2.3. Treatment Protocol

A standardized treatment regimen was followed. Patients received intravenous hydrocortisone at an initial dose of 3.5 mg/kg for five days, tapered to 1.5 mg/kg for a further three days. Tympanostomy tubes were inserted in cases of concomitant secretory otitis. If hearing recovery was insufficient after intravenous therapy, intratympanic dexamethasone (4 mg/mL) was administered for three days. Antiviral medication was not used. Upon discharge from the hospital, patients were prescribed a regimen of vestibular rehabilitation exercises to facilitate recovery. MRI was performed for all patients with persistent hearing asymmetry to exclude retrocochlear pathology. Individuals experiencing recurrent vertigo were excluded from the final analysis.

### 2.4. Statistical Analysis

All statistical analyses were performed using R software (version 4.5.0; R Foundation for Statistical Computing). Continuous variables are presented as mean ± standard deviation or median with interquartile range (IQR Q1–Q3), based on their distribution. Normality was assessed using the Shapiro–Wilk test and visual inspection of histograms and Q–Q plots. Categorical variables are summarized as counts and percentages.

The annual frequency of hospital-diagnosed labyrinthitis cases from 2014 to 2024 was analyzed as count data. The observation period for 2024 was adjusted to 0.39 years. A Poisson regression model was used to compare the mean annual case counts between the pre-2020 (2014–2019) and post-2020 (2020–2024) periods, with results reported as case rate ratios (CRR) and 95% confidence intervals (CI).

Changes in hearing thresholds were evaluated using the average air-conduction (AC) pure-tone thresholds at 500, 1000, 2000, and 4000 Hz, expressed as PTA. The difference between baseline and follow-up PTA was defined as the difference between the 3-month follow-up and baseline PTA (ΔPTA = PTAfollow-up − PTAbaseline), where negative values indicate improvement.

The mean ΔPTA was compared between the pre- and post-2020 periods using Welch’s *t*-test.

Clinically important hearing improvement was defined a priori as a ≥10 dB or ≥15 dB reduction in PTA from baseline. Proportions of patients achieving these thresholds were compared between periods using Fisher’s exact test.

Hearing outcomes at follow-up were categorized according to the World Health Organization (WHO) guidelines as: normal/mild (<35 dB HL), hearing-aid candidate (35–79 dB HL), and deaf/profound (≥80 dB HL) [13]. Differences in categorical hearing outcomes between periods were analyzed with the Fisher–Freeman–Halton exact test.

To identify predictors of persistent hearing impairment (defined as being in the hearing-aid candidate or deaf/profound category at follow-up), a multivariable logistic regression model was fitted. The model included baseline PTA (per 5 dB increase), age, sex, and study period (pre- vs. post-2020) as covariates. Model performance was evaluated by assessing discrimination (area under the receiver operating characteristic curve, AUC) and calibration (Hosmer-Lemeshow test and Brier score). The assumption of linearity for continuous predictors was verified by testing log-transformed terms.

A two-sided *p*-value of less than 0.05 was considered statistically significant.

## 3. Results

### 3.1. Patient Characteristics and Study Flow

**Figure 1:**
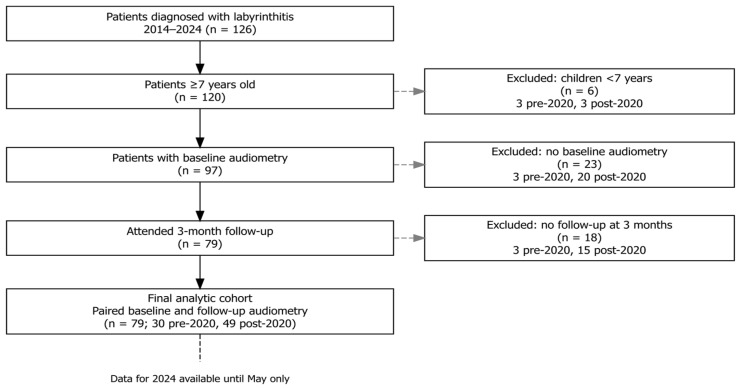
Flowchart of patient exclusions.

The study included 126 patients diagnosed with acute labyrinthitis, treated between January 2014 and May 2024. After excluding 6 children under 7 years of age (who could not undergo routine audiometric testing) and 23 patients lacking baseline audiometry, 97 patients remained. Of these, 79 patients (30 treated before 2020 and 49 treated after 2020) completed the 3-month follow-up and formed the final analytical cohort (Figure 1).

The complete cohort consisted of 46 males (36.5%) and 80 females (63.5%) with a mean age of 47.79 ± 20.16 years (range 1—84 years). The analytical sample (*n* = 79) had comparable gender distribution but was slightly older (mean age 52.0 ± 14.4 years) and included a higher proportion of inpatients (59.5% vs. 44.4% in the complete cohort) (Table 1).

A comparison of baseline characteristics between included patients and those lost to follow-up (*n* = 47) is provided in Supplementary Table S1. The groups were comparable in terms of sex distribution (*p* = 0.3). However, non-included patients were significantly younger (median 42 vs. 51 years, *p* = 0.008), had milder baseline hearing loss (median AC average 31 dB vs. 55 dB, *p* < 0.001), and were more likely to be diagnosed in the post-2020 period (81% vs. 62%, *p* = 0.030). A Cochran-Armitage trend test indicated a non-significant decrease in inclusion rates over the study period (*p* = 0.124; Supplementary Table S2 and Supplementary Figure S1), suggesting that the higher loss to follow-up post-2020 was likely due to pandemic-related healthcare disruptions rather than a systematic selection bias.

### 3.2. Temporal Trends in Labyrinthitis Cases

**Figure 2:**
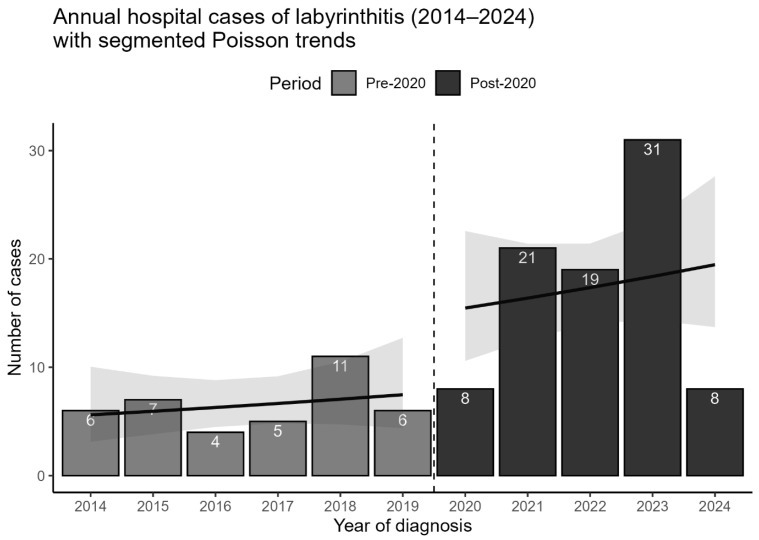
Annual hospital cases of labyrinthitis (2014–2024). Bar heights indicate yearly patient numbers (*n* = 126), with exact counts displayed. Pre-2020 and post-2020 cases are distinguished by shading, with a dashed vertical line marking the separation. Segmented Poisson regression trends with 95% confidence intervals show distinct patterns for each period. Data for 2024 were available only until 23 May.

A marked increase in labyrinthitis cases was observed following 2020. Between 2014 and 2019, 39 patients with labyrinthitis were diagnosed at our center (mean 6.5 cases/year), compared to 87 patients diagnosed between January 2020 and May 2024 (mean 9.8 cases/year). Poisson regression confirmed a significantly higher case rate in the post-2020 period (CRR = 3.05, 95% CI 2.07–4.57, *p* < 0.001) (Table 2). A segmented regression analysis demonstrated a clear upward shift in case numbers after 2020, with no significant trend within the pre-2020 period (Figure 2).

### 3.3. Hearing Recovery Patterns

**Figure 3:**
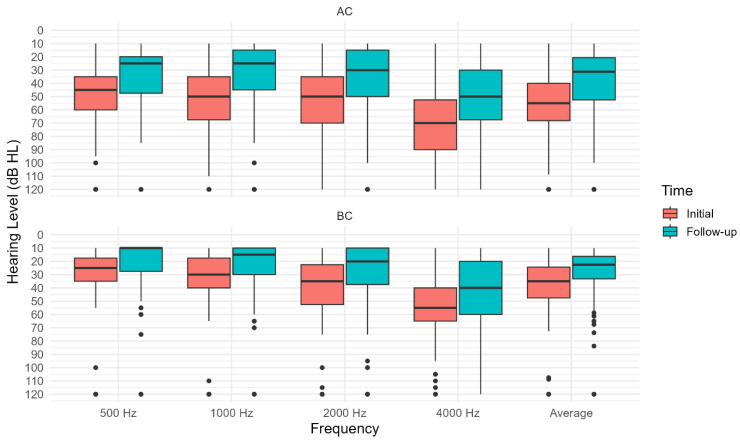
Comparison of AC (**top**) and BC (**bottom**) thresholds before and at follow-up across different frequencies. AC and BC average is the average value through all frequencies.

Significant hearing improvement occurred between baseline and 3-month follow-up across all tested frequencies. AC thresholds improved substantially, with median gains of 15.0 dB at individual frequencies (500, 1000, 2000, and 4000 Hz) and an overall median improvement of 13.8 dB (IQR 0.0–28.8). Bone conduction (BC) thresholds showed more modest but consistent improvement, with median gains ranging from 0.0 dB to 10.0 dB across frequencies and an overall median improvement of 7.5 dB (IQR 0.0–15.0) (Figure 3).

### 3.4. Hearing Outcomes and Recovery Patterns Before and After 2020

Although the post-2020 period saw a significant increase in labyrinthitis case numbers, the analysis of hearing outcomes revealed no substantial differences between the two eras.

At the 3-month follow-up, the overall improvement in hearing thresholds was substantial in both study periods (Table 3). The mean change in pure-tone average (ΔPTA) from baseline to follow-up was −16.7 ± 15.5 dB in patients diagnosed before 2020 and −15.3 ± 17.7 dB after 2020. The mean difference between periods was small and not statistically significant (Δ = 1.3 [95% CI: −8.9 to 6.2]; *p* = 0.73, Welch’s *t*-test).

Clinically important improvement, defined as ≥10 dB, occurred in 19 of 30 (63.3%) patients in the pre-2020 period and in 30 of 49 (61.2%) patients in the post-2020 period. For the stricter ≥15 dB criterion, improvement was achieved in 53.3% and 46.9% of patients, respectively (Fisher’s exact *p* = 0.65).

Audiometric thresholds were comparable between the groups at both baseline and follow-up. Baseline AC thresholds in affected ears measured a median of 51.3 dB (IQR 40.0–67.5) pre-2020 versus 56.3 dB (IQR 42.5–67.5) post-2020 (*p* = 0.571). Similarly, at the 3-month follow-up, AC thresholds showed comparable improvement in both periods (median 28.8 dB (IQR 19.7–49.7) vs. 31.3 dB (IQR 21.3–52.5), *p* = 0.606). BC thresholds followed an equivalent pattern *(*Table 4).

The distribution of final hearing categories also showed no statistically significant variation (*p* = 0.636). Normal hearing or mild impairment was achieved by 56.7% of pre-2020 patients compared to 55.1% of post-2020 patients, while the proportions of patients classified as needing hearing-aid (36.7% vs. 30.6%) and deaf/profound (6.7% vs. 14.3%) were not significantly different (Table 5).

### 3.5. Baseline Hearing as the Primary Determinant of Outcome

Given the clinical observation of potentially more severe cases and the absence of a period effect, we investigated which factors truly predict persistent hearing impairment.

**Figure 4:**
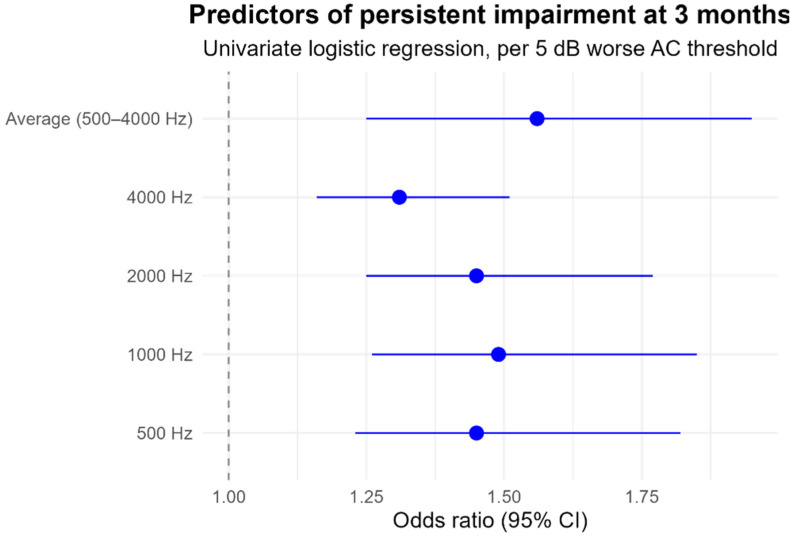
Baseline AC thresholds and their association with persistent hearing impairment in patients with acute labyrinthitis. Higher AC thresholds at baseline were associated with increased odds of persistent hearing loss.

Initial univariate logistic regression analyses demonstrated that baseline AC thresholds at each individual frequency (500, 1000, 2000, and 4000 Hz) were significant predictors of persistent impairment, with odds ratios per 5 dB worsening ranging from 1.31 to 1.49 (Figure 4). As the predictive effects were consistent across frequencies and the thresholds were highly correlated, the average AC across 500–4000 Hz was selected as the primary, more robust predictor for the final multivariable model.

A multivariable logistic regression model, which included this average baseline AC threshold alongside age, sex, and diagnostic period, identified baseline hearing level as the only significant predictor (Table 6). Each 5 dB increase in baseline AC average increased the odds of persistent impairment by 56% (OR 1.56, 95% CI 1.25–1.95, *p* < 0.001). Age, sex, and diagnostic period were not significantly associated with outcome. The model demonstrated excellent discrimination (AUC = 0.87, 95% CI 0.79–0.95) and good calibration (Brier score = 0.144; Hosmer-Lemeshow *p* = 0.529).

As detailed in the Methods, we systematically evaluated potential selection bias due to differential follow-up rates. Patients lost to follow-up (*n* = 47) had significantly milder baseline hearing loss (median 31 dB vs. 55 dB, *p* < 0.001) and were more frequently diagnosed post-2020 (81% vs. 62%, *p* = 0.030). Despite this systematic difference, the consistent hearing recovery patterns observed across all outcome measures—including ΔPTA, clinically important improvement thresholds, final hearing categories, and both AC and BC thresholds—provide robust evidence that our primary conclusions are not substantially influenced by this selection bias. The comparable outcomes across periods, despite the exclusion of predominantly milder cases post-2020, reinforce that the fundamental recovery potential remained unchanged.

### 3.6. Association with COVID-19

An exploratory analysis of SARS-CoV-2 association was performed. Among the 87 patients diagnosed in the post-2020 period, 11 (12.6%) had laboratory-confirmed COVID-19 infection prior to labyrinthitis diagnosis. The median time between a positive COVID-19 test and the diagnosis of labyrinthitis was 21 (IQR 14–107) days. However, 53 patients (60.9%) had unknown SARS-CoV-2 status due to limited testing availability and accessibility constraints during the pandemic period. Consequently, no reliable analysis regarding the association between COVID-19 and hearing outcomes could be performed due to this substantial missing data and the lack of a proper control group.

## 4. Discussion

The primary objective of this study was to evaluate the hypothesis that the incidence of labyrinthitis increased following the onset of the COVID-19 pandemic compared with the preceding years. The analysis was based on hospital case counts, specifically on the annual number of patients treated, rather than population-based incidence data. Consequently, the findings should be interpreted as representing an institutional trend, which may partly reflect alterations in referral patterns or healthcare-seeking behavior during and after the COVID-19 pandemic. However, despite this methodological limitation, the observed pattern strongly suggests a true epidemiological shift, as the increase persisted beyond 2020 and was most pronounced during 2021, coinciding with the Delta variant wave.

In December 2019, the emergence of COVID-19 triggered a global health crisis, affecting millions of people worldwide. Clinical symptoms typically develop within 2–14 days of exposure, and most commonly include fever, cough, and shortness of breath, with frequent disturbances of smell and taste. Infection with SARS-CoV-2 can also result in long-term, multisystem complications, collectively referred to as post-acute sequelae of COVID-19 (long COVID), affecting multiple organ systems and highlighting the sustained impact of the disease [7,14]. Our findings indicate a significant increase in the incidence of labyrinthitis in the period following the onset of the COVID-19 pandemic. When comparing the two-time frames, only 39 cases were documented prior to 2020, whereas 87 cases were recorded from 2020 onward. The most pronounced annual rise was observed in 2021, while the lowest incidence occurred in 2016. This corresponds to approximately a threefold increase in annual case rate after 2020, consistent with international trends reporting similar surges in otologic diagnoses during the pandemic period. While hearing loss was less frequently associated with the Alpha variant, the Delta variant—first detected in 2021—was associated with a higher incidence and greater severity, potentially due to increased inflammation, thrombosis, or direct viral involvement of the auditory system [8]. Notably, research on the Delta variant aligns with our findings, which demonstrated a markedly increased incidence of labyrinthitis in 2021. This shift suggests that the pandemic period may have contributed to an increase in labyrinthitis cases, either directly through viral effects or indirectly via behavioral, environmental, or systemic health factors. Although our study cannot confirm causality, the overlap between the Delta wave and the peak in labyrinthitis strongly supports a biologically plausible connection between SARS-CoV-2 infection and inner ear pathology. Although the precise mechanisms underlying COVID-19-related auditory dysfunction remain unclear, several plausible pathways have been proposed. First, direct viral invasion of the cochlea or vestibular nerve may induce cochleitis or neuritis, resulting in vertigo, tinnitus, or hearing loss. Second, immune cross-reactivity may lead antibodies or T cells to erroneously target inner ear antigens, causing autoimmune-mediated injury. Third, vascular disturbances appear particularly relevant: the cochlea and semicircular canals are highly susceptible to ischemia due to the absence of collateral circulation, and COVID-19-associated coagulopathy or microthrombosis could compromise perfusion of the auditory system. Furthermore, SARS-CoV-2 entry through angiotensin-converting enzyme 2 (ACE2) receptors expressed in cochlear and vestibular tissues provides an additional mechanistic explanation for possible viral tropism in the inner ear. The expression of ACE2 in vascular smooth muscle further indicates potential viral entry points, increasing the risk of thrombotic or ischemic injury. Dysregulated immune responses with excessive cytokine release may exert additional cytotoxic effects on the inner ear. Finally, impaired oxygenation of auditory tissues, given the inner ear’s high metabolic demand, may also contribute to sensorineural hearing loss in COVID-19 patients [8,9,10,11]. Recent medical literature has increasingly reported cases of labyrinthitis following COVID-19, with hearing loss frequently emerging within weeks of SARS-CoV-2 infection. In our study, hearing loss was typically diagnosed approximately 21 days after a positive COVID-19 test. This delayed onset of labyrinthitis after COVID-19 infection may hypothetically be attributed primarily to immune-mediated mechanisms rather than direct viral replication in the inner ear [12,15,16,17]. Such a latency period supports the concept of post-infectious autoimmunity or cytokine dysregulation, mirroring the inflammatory profile observed in long COVID.

Persistent dysregulation of the immune response, including prolonged cytokine activity, could play a central role. As reported in the medical literature, patients with long COVID frequently exhibit a pro-inflammatory cytokine profile, characterized by elevated IL-2, IL-17, and TNF-α, alongside reduced IL-4 and IL-10. Similarly, increased levels of IL-1β and other pro-inflammatory cytokines have been documented in individuals with sudden hearing loss [18,19]. This overlap suggests shared immunopathological mechanisms between COVID-19–related labyrinthitis and idiopathic sudden hearing loss.

Moreover, SARS-CoV-2 infection has been associated with a high incidence of de novo autoimmunity and, in patients with pre-existing autoimmune conditions, with the onset of additional autoimmune diseases or exacerbation of symptoms [14]. It is therefore plausible that immune cross-reactivity causes antibodies or T cells activated during infection to erroneously target inner ear antigens, resulting in autoimmune-mediated labyrinthitis and potentially explaining why symptoms often manifest only after viral clearance [9].

Additional mechanisms—such as microvascular dysfunction, thrombotic events compromising cochlear perfusion, post-infectious inflammatory cascades, or reactivation of latent viruses—may also contribute to late-onset audiovestibular complications [1,2,9]. Taken together, these observations support the hypothesis that delayed labyrinthitis or hearing loss following COVID-19 is predominantly mediated by persistent immune and vascular processes rather than by direct viral invasion. Our findings therefore complement emerging global data supporting the concept of post-viral immune-mediated inner ear pathology as a key consequence of SARS-CoV-2 infection. The potential contribution of ototoxic medications administered during the pandemic should also be considered. Drugs such as chloroquine and hydroxychloroquine, which were used in some countries for COVID-19 treatment, are known to possess ototoxic potential: chloroquine is associated with sudden-onset auditory effects, whereas hydroxychloroquine more commonly induces ototoxicity after prolonged use, with hearing loss reported to occur anywhere from one month to several years [20]. The doses employed for COVID-19 treatment were frequently higher than those used for malaria, further increasing the risk of adverse auditory outcomes. Although data on ototoxic medication exposure were unavailable in our cohort, such confounding factors cannot be excluded and may have contributed to individual variability in hearing recovery [21,22]. Several case reports have documented sudden sensorineural hearing loss (SSNHL) as a rare otolaryngologic adverse event following COVID-19 vaccination. Although a causal relationship has not been definitively established, these observations highlight the need for ongoing pharmacovigilance and further investigation into possible immunological mechanisms [11]. However, vaccination status was not systematically recorded in our dataset, limiting our ability to explore potential associations with auditory outcomes. Finally, during the early phase of the pandemic, patients were routinely tested for COVID-19; however, in subsequent years, systematic testing was no longer consistently performed due to isolation requirements and the adoption of home-based care. Within our cohort, some patients were tested using polymerase chain reaction (PCR), while others underwent rapid antigen assays, which differ in sensitivity and reliability. Moreover, COVID-19 status cannot be determined retrospectively once the acute infection has resolved. Consequently, the lack of systematic testing and the high proportion of unknown infection status (60.9% of post-2020 cases) represent important methodological limitations that constrain causal interpretation. This shift in testing practices, combined with the typically delayed onset of labyrinthitis following recovery from COVID-19, likely contributed to underreporting and explains why many patients tested negative at the time of presentation or were not tested at all. Nevertheless, the marked increase in incidence observed after 2020 coincides with the pandemic period. In addition to these epidemiological trends, audiometric data were analyzed to further characterize hearing outcomes within the cohort. Despite a marked post-2020 increase in case numbers, hearing recovery remained comparable across study periods, suggesting that pandemic-related disruptions did not significantly affect auditory prognosis. Complete audiometric data were available for 79 patients, while results for the remaining 47 were unavailable, likely due to testing conducted at external institutions. During the COVID-19 pandemic, additional factors such as isolation measures and restricted access to healthcare may have further contributed to incomplete audiological documentation. At follow-up, both AC and BC thresholds demonstrated meaningful improvement. This pattern indicates partial yet clinically significant recovery of cochlear function, aligning with previous reports of reversible immune- or infection-mediated inner ear injury [17,23,24]. Although the incidence of labyrinthitis increased following the onset of the COVID-19 pandemic, our comparative analysis of audiometric outcomes before and after 2020 did not demonstrate significant differences in hearing recovery between the two groups. These results suggest that, while the pandemic period may have affected case frequency, it did not appear to influence overall auditory prognosis. The consistency of audiometric outcomes across both time periods further supports the conclusion that baseline hearing status—rather than external or temporal factors—remains the principal determinant of recovery potential. In our cohort, hearing outcomes at the 3-month follow-up were largely comparable regardless of whether patients were diagnosed before or after 2020. In both periods, more than half of the patients achieved normal or mild hearing levels, and the proportion of hearing-aid candidates remained similar. Although the post-2020 group showed a higher percentage of deafness (14.3% vs. 6.7%), this difference did not reach statistical significance. Overall, these findings indicate that despite potential shifts in clinical practice or external influences over time, the short-term auditory prognosis in acute labyrinthitis remained stable. This stability may suggest that the underlying pathophysiological mechanisms—whether immune-mediated, vascular, or post-infectious—affect auditory outcomes in a comparable manner regardless of the pandemic context. It also indicates that timely application of standardized therapeutic protocols remains effective in preserving auditory function, even amid the increased case burden during COVID-19. A notable limitation, however, is the lack of precise data on the interval between symptom onset and treatment initiation; although therapy was consistently standardized, this information was not routinely recorded and could not be reliably reconstructed retrospectively. Our findings indicate that baseline hearing level is a key determinant of long-term auditory outcomes. Patients with normal baseline hearing were largely protected from persistent impairment, highlighting the prognostic value of early functional integrity of the auditory system. The absence of significant associations with demographic factors such as age or sex further underscores that initial hearing status outweighs these variables in predicting recovery. The observed 56% increase in risk for every 5 dB decrement in baseline thresholds suggests a dose–response relationship, emphasizing the clinical importance of stratifying patients at presentation. These results support the notion that the severity of early hearing loss reflects the extent of underlying cochlear or neural injury, which constrains the potential for full recovery. Accordingly, baseline audiometric assessment should serve not only as a diagnostic tool but also as a prognostic guide, informing follow-up strategies and patient counseling. Having established the functional outcomes, we next examined demographic factors, beginning with age, to assess potential correlations with labyrinthitis incidence.

The mean age of patients in our cohort was 47.79 years, with a range of 1–84 years, confirming that labyrinthitis can affect individuals across the lifespan. Nevertheless, our findings are consistent with prior reports indicating a higher prevalence among adults aged 30 to 60 years [1]. Studies also report a predominance of cases within the 30 to 50-year age group [25]. The inclusion of both pediatric and elderly patients suggests that, although less frequently reported, labyrinthitis can occur outside the commonly cited adult age range. This broader age distribution may reflect regional epidemiological differences, improved diagnostic sensitivity, or variation in healthcare-seeking behavior. Collectively, these observations reinforce the importance of considering labyrinthitis in the differential diagnosis for patients of all ages and provide a foundation for examining other demographic factors, such as gender, that may influence susceptibility.

Consistent with these age-related trends, 63.5% of patients in our cohort were female, compared with 36.5% male, supporting the observation that labyrinthitis appears more prevalent among women. This finding aligns with prior research on vestibulocochlear disorders, which consistently shows that females are disproportionately affected by vestibular dysfunctions, including labyrinthitis. Several studies report a general 2:1 female-to-male ratio in the prevalence of specific vestibular conditions. This gender disparity is thought to be influenced by hormonal factors and other physiological differences, making women more susceptible to conditions such as labyrinthitis [26,27]. These demographic insights carry practical implications for clinical management, as recognition of higher susceptibility among specific patient groups can facilitate early diagnosis, targeted monitoring, and individualized interventions. Unfortunately, data on certain potential confounders, such as autoimmune diseases, smoking history, and detailed ototoxic medication exposure, were not systematically available, which may limit the comprehensive assessment of all risk factors for labyrinthitis.

While no cases of labyrinthitis ossificans were observed in our cohort—consistent with its association with suppurative infections rather than idiopathic inflammation—this potential late sequela remains relevant for the small subset of patients who progress to profound hearing loss. In such cases, cochlear implantation may be considered, and preoperative recognition of ossification is crucial as it can obscure cochlear landmarks and complicate electrode array placement. Advanced imaging and specialized surgical techniques may be required to navigate these challenges [28].

At the UKC MB, patients diagnosed with acute labyrinthitis were treated according to a standardized institutional protocol, with therapy adjusted to the clinical presentation and presumed etiology. In cases of viral labyrinthitis, management generally included symptomatic treatment with antivertigo medication, corticosteroids in selected cases, and nasal decongestants, whereas bacterial labyrinthitis was managed with antibiotic therapy directed toward the suspected or confirmed infectious source.

This approach is broadly consistent with current expert guidelines, which advocate for etiologic and symptom-oriented individualization of therapy rather than the routine administration of antibiotics, as further discussed in continuation.

In cases of viral labyrinthitis, conservative outpatient management—including rest, hydration, and vigilant monitoring for neurological red flags such as diplopia, weakness, or gait disturbances—is generally recommended. The routine use of antivirals or corticosteroids remains unsupported by robust evidence [1]. Nonetheless, selected case reports of COVID-19–associated labyrinthitis have documented successful outcomes with conservative therapy, occasionally supplemented with corticosteroids [10,29]. In bacterial labyrinthitis, antibiotic therapy should be guided by the source: oral antibiotics are appropriate for uncomplicated otitis media, whereas intravenous therapy is warranted if meningitis is suspected or if the infection fails to respond to initial treatment [1,30]. The use of vestibular suppressants aligns with symptomatic relief strategies; however, prolonged administration beyond 72 h may hinder vestibular compensation, which is essential for recovery [1]. Early mobilization, even in the presence of vertigo, is recommended, as evidence indicates it facilitates vestibular adaptation and improves functional outcomes [1].

For patients presenting with SSNHL, early corticosteroid administration may be beneficial, particularly in idiopathic or suspected autoimmune cases. Such decisions are best made under specialist supervision [1,15].

Persistent residual symptoms such as tinnitus or balance disturbances may negatively impact quality of life. Prompt referral for vestibular rehabilitation or tinnitus management strategies—such as tinnitus retraining therapy, hearing aids or biofeedback—is recommended [1,31].

In summary, the standardized protocol at UKC MB ensured consistent management, while tailoring therapy to likely etiology aligns with international guidelines. Further refinement toward even more individualized treatment strategies may optimize outcomes, minimize unnecessary interventions, and reinforce adherence to best-practice recommendations.

## 5. Conclusions

Our study demonstrates a clear increase in the incidence of labyrinthitis following the onset of the COVID-19 pandemic, with the majority of cases occurring after 2020. Despite this rise in incidence, our analysis of audiometric outcomes revealed no significant differences in hearing recovery or functional prognosis between patients diagnosed before and after 2020. Hearing outcomes at 3 months remained comparable before and after 2020, with stable proportions of recovery, hearing-aid use, and profound loss. Baseline hearing level emerged as the key prognostic factor, where each 5 dB decrement increasing long-term risk, while age and sex showed no effect.

These findings suggest that although the pandemic period may have influenced the frequency of labyrinthitis presentations, the underlying pathophysiological mechanisms and treatment outcomes remained consistent. Demographic analysis further confirmed that labyrinthitis can occur across all age groups but is most common in middle-aged adults and more frequently affects women, reflecting previously reported epidemiological patterns.

This study has several limitations. Our analysis was based on hospital case counts, not true population incidence. Therefore, results should be interpreted as an institutional trend, which may partly reflect changes in referral patterns or healthcare-seeking behavior during and after the COVID-19 pandemic. The sample size was relatively small, which limited the statistical power of subgroup analyses and increased the risk of wide confidence intervals in regression models. Second, the retrospective design and reliance on routinely collected clinical data introduced the possibility of misclassification and incomplete information. COVID-19 status was not systematically tested in all patients during the early stages of the pandemic, which precluded robust assessment of the temporal relationship between SARS-CoV-2 infection and labyrinthitis outcomes. Third, audiometric follow-up was available for only 63% of eligible patients, which may have introduced selection bias. Those lost to follow-up were generally younger and had milder initial hearing loss (Supplementary Table S1), suggesting that our analytical cohort may overrepresent severe cases. The notably higher rate of missing follow-up after 2020 (Supplementary Figure S1) likely stems from pandemic-related barriers to healthcare access rather than from a change in the disease itself. Finally, the 3-month follow-up period, while capturing the critical window for initial hearing recovery and aligning with standard endpoints used in SSNHL studies [32,33,34], does not inform on long-term auditory outcomes beyond one year or the potential development of very late sequelae. Although literature suggests that most hearing recovery occurs within the first 3 months, we have tempered our conclusions accordingly to avoid overstating long-term prognostic claims.

These limitations should be considered when interpreting the findings, and further prospective studies with larger cohorts are warranted to validate our observations. On the other hand, a major strength lies in the standardized diagnostic and therapeutic approach applied to all patients, ensuring consistency of evaluation and management.

From a clinical perspective, early audiometric assessment provides crucial prognostic information, and the delivery of standardized, etiology-driven treatment protocols to optimize patient outcomes.

## Figures and Tables

**Table 1:** Demographic and clinical characteristics of patients diagnosed with acute labyrinthitis.

		All Cases (N = 126)		Sample (N = 79)	
		N (%)		N (%)	
gender	male	46 (36.5)		26 (32.9)	
	female	80 (63.5)		53 (67.1)	
COVID-19 positive		11 (8.7)		7 (8.9)	
type of care	outpatient	70 (55.6)		32 (40.5)	
	inpatient	56 (44.4)		47 (59.5)	
		mean ± SD	min–max	mean ± SD	min–max
age		47.8 ± 20.2	1–84	52.0 ± 14.4	8–81

**Table 2:** Annual case counts of labyrinthitis before and after 2020. The table shows the number of patients diagnosed with labyrinthitis at our center during the pre-2020 (2014–2019) and post-2020 (2020–May 2024) periods. Mean annual case counts and CRR were estimated using Poisson regression. Data for 2024 were available until 23 May, and exposure time for that year was adjusted to 0.39 years. Results reflect hospital case counts, not population incidence.

Period	Years Observed	Total Cases	Mean Cases/Year	Case Rate Ratio (95% CI)	*p*-Value
Pre-2020	6 (2014–2019)	39	6.5	Reference	–
Post-2020	4.39 (2020–May 2024)	87	19.8	3.05 (2.07–4.57)	<0.001

**Table 3:** Change in pure-tone average (ΔPTA, dB HL) between baseline and 3-month follow-up by period.

Period	n	Mean ± SD (ΔPTA dB)	95% CI (ΔPTA)	Improved ≥ 10 dB	Improved ≥ 15 dB
Pre-2020	30	−16.7 ± 15.5	−22.5 to −10.9	19 (63.3%)	16 (53.3%)
Post-2020	49	−15.3 ± 17.7	−20.4 to −10.2	30 (61.2%)	23 (46.9%)
Comparison (Post vs. Pre)		Δ = 1.3 [95% CI: (−8.9 to 6.2)]	*p* = 0.726	*p* = 1.000	*p* = 0.647

ΔPTA < 0 indicates hearing improvement. Clinically important improvement defined as ≥10 dB and ≥15 dB thresholds. Welch’s *t*-test used for the difference in means; Fisher’s exact test used for differences in proportions.

**Table 4:** Comparison of hearing outcomes in patients treated before and after 2020.

	Before 2020		2020 and After		
	median (Q1–Q3)	min–max	median (Q1–Q3)	min–max	*p*-value
Average AC affected ear	51.3 (40.0– 67.5)	17.5–120.0	56.25 (42.5–67.5)	10.0–120.0	0.571
Average AC healthy ear	15.6 (11.3–20.9)	10.0–63.8	18.8 (12.5–30.0)	10.0–51.3	0.225
Average AC affected ear control	28.8 (19.7–49.7)	10.0–120.0	31.3 (21.3–52.5)	10.0–120.0	0.606
Average BC affected ear	30 (23.4–46.6)	11.3–120.0	36.3 (26.3–50.0)	10.0–120.0	0.422
Average BC healthy ear	12.5 (10.3–17.1)	10.0–55.0	13.8 (11.3–22.5)	10.0–48.8	0.290
Average BC affected ear control	22.5 (15.3–31.3)	10.0–120.0	22.5 (17.5–36.3)	10.0–120.0	0.482

**Table 5:** Hearing outcomes at 3-month follow-up stratified by period (2014–2024). Values are n (%). Between-group comparison was performed using Fisher–Freeman–Halton exact test (*p* = 0.636).

Period	Normal/Mild	Hearing-Aid Candidate	Deaf/Profound	*p*-Value
Pre-2020	17 (56.7%)	11 (36.7%)	2 (6.7%)	0.636
Post-2020	27 (55.1%)	15 (30.6%)	7 (14.3%)	

**Table 6:** Logistic regression analysis of predictors of impaired hearing at 3-month follow-up (AC average per 5 dB).

Predictor		Estimate (SE)	OR (95% CI)		*p*-Value
(Intercept)		−6.011 (1.729)	0.002 [0–0.054]		0.001
Baseline AC average (per 5 dB)		0.448 (0.114)	1.565 [1.293–2.029]		<0.001
Age at diagnosis (per year)		0.018 (0.022)	1.018 [0.976–1.064]		0.414
Male sex (ref = female)		−0.155 (0.656)	0.857 [0.235–3.178]		0.814
Post-2020 (ref = Pre-2020)		−0.235 (0.616)	0.79 [0.229–2.648]		0.702
Model performance: Brier score = 0.144; AUC = 0.87 (95% CI 0.79–0.95); Hosmer–Lemeshow *p* = 0.529.					
Period	*n*	Mean ± SD (ΔPTA dB)	95% CI (ΔPTA)	Improved ≥10 dB	Improved ≥15 dB
Pre-2020	30	−16.7 ± 15.5	−22.5 to −10.9	19 (63.3%)	16 (53.3%)
Post-2020	49	−15.3 ± 17.7	−20.4 to −10.2	30 (61.2%)	23 (46.9%)
Comparison (Post vs. Pre)		Δ = 1.3 [95% CI: (−8.9 to 6.2)]	*p* = 0.726	*p* = 1.000	*p* = 0.647
ΔPTA < 0 indicates hearing improvement. Clinically important improvement defined as ≥10 dB and ≥15 dB thresholds. Welch’s *t*-test used for the difference in means; Fisher’s exact test used for differences in proportions.					

## Data Availability

The datasets generated and/or analyzed during the current study are not publicly available due to patient confidentiality and the sensitive nature of the clinical data but are available from the corresponding author upon reasonable request. Requestors will be required to submit a methodologically sound proposal and sign a data access agreement.

## Supplementary Materials

The following supporting information can be downloaded at: https://www.mdpi.com/article/10.3390/medicina61122083/s1, Figure S1: Yearly distribution of included vs. non-included patients with labyrinthitis (2014–2024); Table S1: Comparison of included vs. non-included patients; Table S2: Cochran–Armitage trend test for annual inclusion rates (2014–2024).

## Abbreviations

The following abbreviations are used in this manuscript:

ΔPTA	mean change in pure-tone average
AC	air conduction
ACE2	angiotensin-converting enzyme 2
AUC	area under the receiver operating characteristic curve
BC	bone conduction
CI	confidence interval
CMV	cytomegalovirus
COVID-19	coronavirus disease 19
CRR	case rate ratio
dB	decibel
e. g.	exempli gratia (for example)
HIV	human immunodeficiency virus
HL	hearing loss
Hz	Hertz
ICD-10	International Classification of Diseases, Tenth Revision
IQR	interquartile range
MRI	magnetic resonance imaging
OR	odds ratio
PCR	polymerase chain reaction
PTA	pure-tone average
RSV	respiratory syncytial virus
SARS-CoV-2	severe acute respiratory syndrome coronavirus 2
SSNHL	sudden sensorineural hearing loss
UKC MB	University Clinical Center Maribor
WHO	World Health Organization
