# Supplementary material for: Comparison of Audiometric Outcomes Following Acute Labyrinthitis
Source: Medicina (Kaunas). 2025 Nov 22;61(12):2083. doi: 10.3390/medicina61122083 (PMC12734768; doi:10.3390/medicina61122083)
Supplement: Supplementary file 1 [file medicina-61-02083-s001.zip › Supplementary table S1.pdf]

*Supplementary Table S1. Comparison of included vs. non-included patients.**

Characteristic	Not included N = 47[1]	Included N = 79[1]	p-value[2]
**Sex (male %)**			0.3
Female	20 (43%)	26 (33%)	
Male	27 (57%)	53 (67%)	
**Age (years)**	42 (22–62)	51 (43–63)	0.008
**Post-2020 period**			0.030
Pre-2020	9 (19%)	30 (38%)	
Post-2020	38 (81%)	49 (62%)	
**Baseline AC average (dB HL)**	31 (23–38)	55 (40–69)	<0.001

[1]Continuous variables are shown as median (IQR) and compared with the Mann–Whitney U test; categorical variables as n (%) and compared with Fisher's exact test.

[2]Fisher's exact test; Wilcoxon rank sum test
