# Supplementary material for: Comparison of Audiometric Outcomes Following Acute Labyrinthitis
Source: Medicina (Kaunas). 2025 Nov 22;61(12):2083. doi: 10.3390/medicina61122083 (PMC12734768; doi:10.3390/medicina61122083)
Supplement: Supplementary file 1 [file medicina-61-02083-s001.zip › Supplementary table S2.pdf]

Supplementary Table S2. Cochran–Armitage trend test for annual inclusion rates (2014–2024).

Test	Z statistic	p value	Direction	Interpretation
Cochran–Armitage trend	-1.54	0.124	Decrease	Nonsignificant downward trend
