# Supplementary material for: Comparison of Audiometric Outcomes Following Acute Labyrinthitis
Source: Medicina (Kaunas). 2025 Nov 22;61(12):2083. doi: 10.3390/medicina61122083 (PMC12734768; doi:10.3390/medicina61122083)
Supplement: Supplementary file 1 [file medicina-61-02083-s001.zip › Supplementary_figure_S1.pdf]

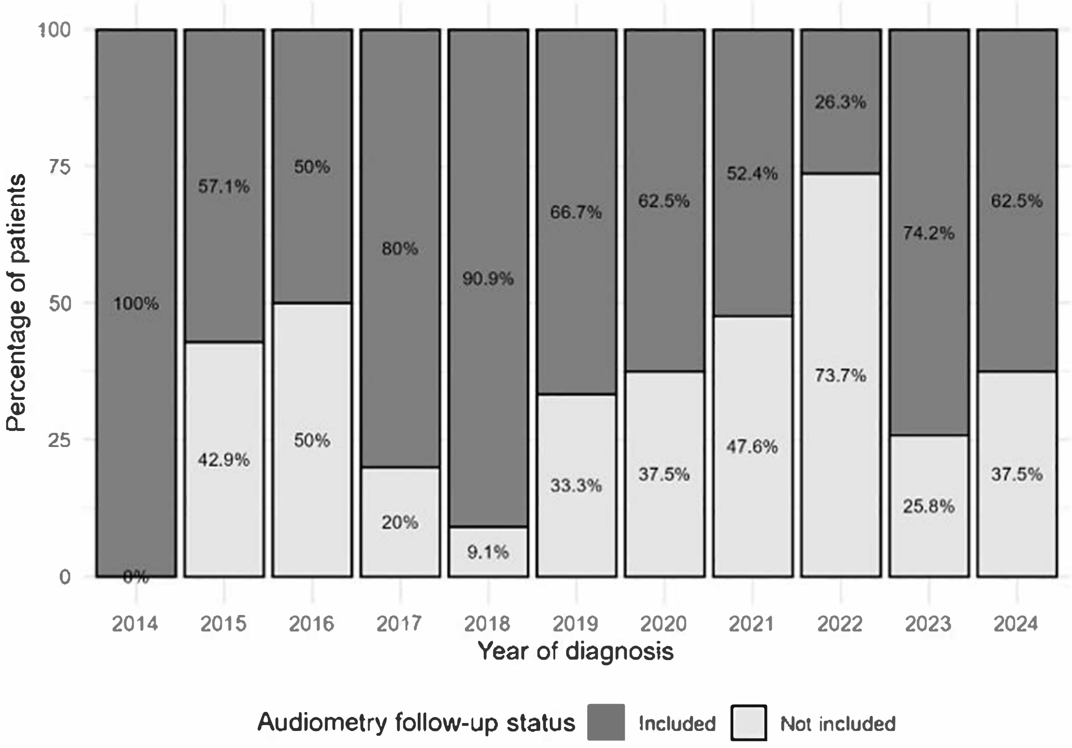

Supplementary Figure S1. Yearly distribution of included vs non-included patients with labyrinthitis (2014–2024)
